# Intermittent injection of Methylprednisolone Sodium Succinate in the treatment of Cervical Spinal Cord injury complicated with incomplete paraplegia

**DOI:** 10.12669/pjms.35.1.211

**Published:** 2019

**Authors:** Wei Wang, Baoshu Zuo, Haixia Liu, Limin Cui

**Affiliations:** 1 Wei Wang, Binzhou People’s Hospital, Binzhou, Shandong-256610, China; 2 Baoshu Zuo, Binzhou People’s Hospital, Binzhou, Shandong-256610, China; 3 Haixia Liu, Binzhou People’s Hospital, Binzhou, Shandong-256610, China; 4 Limin Cui, Binzhou People’s Hospital, Binzhou, Shandong-256610, China

**Keywords:** Incomplete paraplegia, Methylprednisolone sodium Succinate, Spinal cord injury

## Abstract

**Objective:**

To evaluate the clinical efficacy and safety of intermittent injection of methylprednisolone sodium succinate in the treatment of cervical spinal cord injury complicated with incomplete paraplegia.

**Methods:**

Seventy-eight patients with cervical spinal cord injury complicated with incomplete paraplegia who were admitted between August 2016 and December 2017 were enrolled and grouped into an observation group and a control group using random number table, 39 in each group. Patients in the control group were given vertebral body decompression and bone grafting and internal fixation according to the severity of spinal cord compression, while patients in the observation group were treated by methylprednisolone sodium succinate in addition to the same treatment as the control group. The clinical efficacy and medicine associated adverse reactions were compared between the two groups.

**Results:**

The cure rate of the observation group was significantly higher than that of the control group (46.2% (18/39) vs. 20.5% (8/39)). After the treatment, the Japanese Orthopaedic Association (JOA) score and American Spinal Cord Injury Association (ASIA) score of the two groups after treatment were significantly higher compared to before treatment, and the scores of the observation group were much higher than those of the control group (P<0.05). The incidence of adverse reactions of the observation and control groups was 15.4% and 17.9% respectively, and the difference was not statistically significant (P>0.05).

**Conclusion:**

Intermittent injection of methylprednisolone sodium succinate has definite efficacy in treating cervical spinal cord injury complicated with incomplete paraplegia, with a low incidence of adverse reactions; hence it is worth promotion.

## INTRODUCTION

Cervical spinal cord injury may lead to complete paralysis and incomplete paralysis; about 50 among one million people have cervical spinal cord injury every year, and 90% of them die on the way to hospital, suggesting a high fatality rate.^1^ Cervical spinal cord injury refers to the injury of the spinal cord caused by direct or indirect external factors, which can induce functional disorder, abnormality and pathological reactions of movement, sensation and anal/bladder sphincters.^2^,^3^ In recent years, the incidence of cervical spinal cord injury has been increasing year by year, which has a serious impact on quality of life of surviving patients.^4^ Cervical spinal cord injury includes not only primary injury, but also secondary injury induced by local inflammatory reaction, hormone induced edema and systemic inflammatory reaction after injury.^5^ According to relevant clinical experience and literature,^6^,^7^ the treatment schemes for cervical spinal cord injury complicated with incomplete paralysis mainly focused on secondary injury because primary injury is featured by sudden onset and rapid progress. Recently whether adrenocortical hormone can be used in the treatment of cervical spinal cord injury and how to use it has become controversial.^8^,^9^ One of the recent experimental study showed that the early use of high-dose glucocorticoids after cervical spine fracture combined with spinal cord injury could reduce or delay secondary injury and improve the prognosis.^10^

The purpose of this study was to explore the clinical efficacy and safety of the use of methylprednisolone succinate in our patients admitted with cervical spine cord injury having incomplete paralysis.

## METHODS

Seventy-eight patients with cervical spinal cord injury complicated with incomplete paraplegia who were admitted to the hospital between August 2015 and December 2017 were selected and divided into an observation and control groups using random number table, 39 each group. The inclusive criteria included satisfying the diagnostic criteria formulated by American Spinal Cord Injury Association^11^, 20~70 years old, have been diagnosed as spinal cord injury by Magnetic Resonance Imaging (MRI), and with single-segment injury. Those who had cervical spinal cord induced pamplegia, long bone fracture or peripheral nerve injury of four limbs, craniocerebral injury or coagulation disorders were excluded. The study protocol has been reviewed and approved by the medical ethical committee, and the patients have signed informed consent.

### Medicine and instruments

Methylprednisolone sodium succinate (batch number: Z03432; 40 mg each; Pfizer Pharmaceuticals Co. Ltd., China) and HD1.5T MRI scanner (GE, USA) were used.

Patients in the control group underwent vertebral body decompression, bone grafting and internal fixation according to the severity of spinal cord compression, while patients in the observation group were treated by methylprednisolone sodium succinate in addition to the same treatment as the control group. Patients who were injured for less than 3 h received intravenous drip of methylprednisolone sodium succinate at a dose of 30 mg/(kg·h) for 15 minutes; after 30 minutes, the dosage was adjusted to 5.4 mg/(kg·h), for 23 hour. Patients who were injured for 3 ~ 8 h received intravenous drip of methylprednisolone sodium succinate at a dose of 30 mg/(kg·h) for 48 hour. The treatment course was one month. Patients in the two groups also received traditional rehabilitation treatment during hospitalization including acupuncture, massage, functional electrical stimulation, hyperbaric oxygen, somatic sensory neuromuscular enhancement technique, turn-over training, wheelchair training, standing and balance bar training and psychological intervention.

The clinical effect was compared between the two groups. The curative effect was evaluated using spinal cord functional state evaluation method (40 points).^12^ An improvement rate of the score higher than 65%, significant relief of cervical spinal cord compression and basic recovery of cervical region and limbs were evaluated as cured. An improvement rate of the score between 35% and 65%, relief or no aggravation of cervical spinal cord compression, relief of pains on the neck, shoulder and back and improvement of limb functions were evaluated as improved. An improvement rate of the score lower than 35%, aggravation of cervical spinal cord compression, pains on the neck, shoulder and back and no improvement of limb functions were evaluated as not cured.

The spinal cord function and movement of the two groups were compared. Cervical spinal cord functions were evaluated using Japanese Orthopaedic Association (JOA) scores (17 points);^13^ higher score indicated better cervical spinal cord functions. The movement condition of the cervical spinal cord was evaluated using American Spinal Cord Injury Association (ASIA) score (100 points).^14^ The incidences of medicine associated adverse reactions were also compared between the two groups.

### Statistical method

Data were statistically analyzed using SPSS 21.0. Normally distributed measurement data were expressed as mean ± standard deviation (SD); comparison between the two groups was performed using independent sample t-test; enumeration data were processed by Chi-square test. P<0.05 indicated difference was statistically significant.

## RESULTS

**Fig.1:**
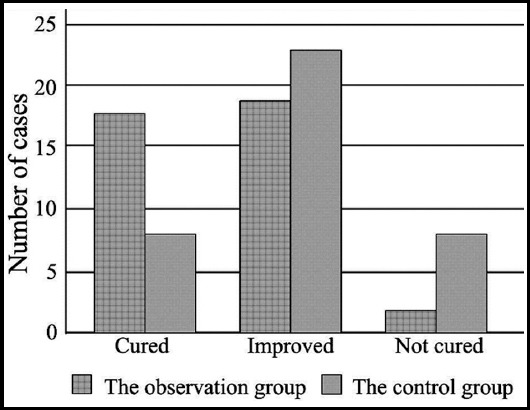
Comparison of clinical effects between the two groups.

The differences of the general data between the two groups had no statistical significance (P>0.05, Table-I). The cure rate of the observation group was higher than that of the control group (46.2% (18/39) vs. 20.5% (8/39)) (X^2^=4.868, P<0.05, Fig.1).

**Table-I:** Comparison of general data between the two groups.

Groups		Observation group	Control group	t/X^2^	P
Sex (M/F)		33/6	31/8	0.216	>0.05
Age (year)		46.8±8.1	47.2±7.9	0.332	>0.05
Heart rate (times·min^-1^)		88.4±11.0	89.7±11.4	0.248	>0.05
Respiratory rate (times·min^-1^)		16.8±3.7	17.3±4.0	0.279	>0.05
Time before treatment (h)	< 3 h	13	11	0.305	>0.05
	3~8 h	26	28	0.319	>0.05
Cause of injury	Fall	11	9	0.301	>0.05
	Traffic accidents	25	28	0.322	>0.05
	Crushing	3	2	0.214	>0.05
Injured segment	C6	25	27	0.327	>0.05
	C7	14	12	0.296	>0.05
	Duration of surgery (min)	85.5±17.7	89.0±27.4	0.174	>0.05

The JOA score and ASIA score of the two groups had no statistically significant differences after treatment (P>0.05). After treatment, the JOA score and ASIA score of the two groups significantly improved. Moreover the scores of the observation group were much higher than those of the control group (P<0.05, Table-II).

**Table-II:** Comparison of JOA and ASIA scores of the two groups before and after treatment.

Group	Observation group	Control group	P
JOA score	17.4±2.3	17.5±2.2	>0.05
	26.9±4.2^*^	21.5±3.4^*^	<0.05
ASIA score	56.47±10.62	55.47±10.88	>0.05
	87.62±6.84^*^	78.17±4.57^*^	<0.05

Note:* indicated P<0.05 compared to before treatment.

In the observation group, there were four cases of urinary tract infection and two cases of pneumonia; the incidence of adverse reactions was 15.4% (6/39). In the control group, there were three cases of urinary tract infection and four cases of pneumonia; the incidence of adverse reactions was 17.9% (7/39). The difference of the incidence of adverse reactions between the two groups had no statistical significance (X^2^=0.917, P>0.05).

## DISCUSSION

Cervical spinal cord injury is usually induced by road traffic accidents, fall from height, violence or sports and has a high incidence among young adults. Cervical spinal cord can result in quadriplegia, respiratory insufficiency and even death.^15^ The pathological changes after spinal cord injury include hemorrhage, necrosis, cyst formation, tissue edema, changes of spinal cord microvessels, ischemia and anoxia.^16^ Surgical decompression and internal fixation can relieve spinal cord edema, reduce spinal cord internal pressure and maintain biomechanical stability to improve blood circulation in spinal cord and avoid or reduce secondary injury of spinal cord; however the efficacy is poor.^17^,^18^

Methylprednisolone sodium succinate, a synthesized steroid hormone, is a commonly used medicine for controlling inflammatory reactions. It can specifically combine with receptors to improve the metabolism of proteins and carbohydrate and has strong anti-inflammatory effect and weak water and sodium retention effects.^19^,^20^ Administration of intermittent high-dose methylprednisolone sodium succinate in the treatment of cervical spinal cord injury can inhibit the occurrence of lipid peroxidation in injured spinal cord,^21^ reduce calcium accumulation and level of lactic acid in cells, improve microcirculation, and prevent ischemia. Intermittent hormone treatment can also achieve inflammatory effect and relieve pain through controlling the release of interleukin. Moreover methylprednisolone sodium succinate is positive in maintaining blood supply of the spinal cord, stabilize cytomembrane and promote the aerobic respiration of cells. An animal model suggested that the application of high-dose methylprednisolone sodium succinate before surgery could effectively inhibit neuro functional impairment.^22^ A clinical study of Wang et al. suggested that methylprednisolone sodium succinate shock therapy could significantly improve the prognosis and neurological function of patients with acute spinal cord injury.^23^

In this study, the patients with cervical spinal cord injury and incomplete paraplegia intermittent injection of methylprednisolone sodium succinate were treated by intermittent injection of methylprednisolone sodium succinate. The results demonstrated that the cure rate of the observation group was 46.2%, which was higher than 20.5% of the control group (P<0.05), and the difference of the incidence of adverse reactions between the two groups had no statistical significance. It indicated that the therapy had definite efficacy. Xu used methylprednisolone sodium succinate shock therapy in the treatment of acute spinal cord injury and found that high dose methylprednisolone sodium succinate shock therapy was effective, safe and reliable in treating acute spinal cord injury,^24^ which was consistent with the finding of this study. The JOA score of the observation group was significantly higher than that of the control group after treatment, indicating that intermittent hormone therapy could effectively reduce pains of patients. In addition, the ASIA score of the observation group after treatment was significantly higher than that of the control group, indicating that intermittent hormone therapy could promote the rehabilitation of patients’ nervous function and restore the motor ability of patients as early as possible, which was in line with the report of Nicolle et al.^25^

### Limitations of the study

This study was single-center, and the sample size was small, which might lead to bias in the research results; therefore our findings need to be verified again in studies with larger sample size.

## CONCLUSION

Intermittent injection of methylprednisolone sodium succinate is effective and safe in treating patients with cervical spinal cord injury and incomplete paraplegia. It can apparently improve the overall effective rate, reduce incidence of adverse reactions, and strengthen the functions of the cervical spinal cord and athletic ability.
